# Insights into emotional wisdom: Older adults’ experiences of emotion and mental health

**DOI:** 10.1192/j.eurpsy.2025.738

**Published:** 2025-08-26

**Authors:** S. von Humboldt

**Affiliations:** William James Center for Research, ISPA – Instituto Universitário, Lisbon, Portugal

## Abstract

**Introduction:**

Emotions wield a substantial influence on mental and physical well-being in old age, encompassing both positive and negative feelings, and effective emotional regulation is linked to aging well. Therefore, understanding the intricate connection between emotions and mental health is essential for promoting healthy aging in late adulthood.

**Objectives:**

This study aims to explore the dimensions between emotions and mental health and the crucial role of emotional wisdom in old age, shedding light on how it influences mental health and overall well-being among older adults.

**Methods:**

This cross-national study included 348 older adults, aged 65 and older, from Portugal and England. Participants’ experiences were portrayed through semi-structured interviews, which were analyzed using content analysis.

**Results:**

Findings revealed four themes: (1) Emotional expression (88.3%), (2) Wisdom acquisition (79.2%), (3) Emotional self-regulation (71.7%), and (4) Ability to calm oneself (68.9%).

**Conclusions:**

Through an exploration of emotional health and wisdom across various cultural lenses, this study illuminated the complex interactions between emotions and mental well-being. It emphasizes the imperative for nuanced understanding and targeted strategies that bridge emotional aging with mental health outcomes, advocating for policies and interventions that foster holistic psychological resilience in older adults.

**Keywords:**

Emotion; emotional regulation; emotional health; emotional wisdom; mental health; older adults.

**Disclosure of Interest:**

None Declared

